# Fatigue Experiment and Failure Mechanism Analysis of Aircraft Titanium Alloy Wing–Body Connection Joint

**DOI:** 10.3390/s25010150

**Published:** 2024-12-30

**Authors:** Xianmin Chen, Shanshan Li, Yuanbo Liang, Shuo Wang, Liang Yan, Shichang Du

**Affiliations:** 1 National Key Laboratory of Strength and Structural Integrity, Aircraft Strength Research Institute of China, Xi’an 710065, China; chenxm007@avic.com (X.C.); liangyuanbo_22@163.com (Y.L.); 2 Department of Industrial Engineering and Management, Shanghai Jiao Tong University, Shanghai 200240, China; sjd8013@sjtu.edu.cn (S.W.); yan-liang@sjtu.edu.cn (L.Y.); lovbin@sjtu.edu.cn (S.D.)

**Keywords:** titanium alloy, wing–body connection joint, flight loading, failure mechanism, structural safety, Paris’ law

## Abstract

Taking the titanium alloy wing–body connection joint at the rear beam of a certain type of aircraft as the research object, this study analyzed the failure mechanism and verified the structural safety of the wing–body connection joint under actual flight loads. Firstly, this study verified the validity of the loading system and the measuring system in the test system through the pre-test, and the repeatability of the test was analyzed for error to ensure the accuracy of the experimental data. Then, the test piece was subjected to 400,000 random load tests of flight takeoffs and landings, 100,000 Class A load tests, and ground–air–ground load tests, and the test piece fractured under the ground–air–ground load tests. Lastly, the mechanism analysis and structural safety verification of the fatigue fracture of the joints were carried out by using a stereo microscope and scanning electron microscope. The results show that fretting fatigue is the main driving force for crack initiation, and the crack shows significant fatigue damage characteristics in the stable growth stage and follows Paris’ law. Entering the final fracture region, the joint mainly experienced ductile fracture, with typical plastic deformation features such as dimples and tear ridges before fracture. The fatigue crack growth behavior of the joint was quantitatively analyzed using Paris’ law, and the calculated crack growth period life was 207,374 loadings. This result proves that the crack initiation life accounts for 95.19% of the full life cycle, which is much higher than the design requirement of 400,000 landings and takeoffs, indicating that the structural design of this test piece is on the conservative side and meets the requirements of aircraft operational safety. This research is of great significance in improving the safety and reliability of aircraft structures.

## 1. Introduction

Titanium alloy is widely used in aircraft structures due to its excellent properties such as high strength, low density, and corrosion resistance. The titanium alloy wing–body connection joint belongs to the main bearing structure of the aircraft. If this important connection structure fails, it will directly lead to catastrophic damage to the aircraft [1]. Statistics show that about 70% of aviation metal structure failure accidents are fatigue failures [2]. Therefore, conducting fatigue experiments on titanium alloy wing–body connection joints and analyzing the failure mechanism is a requirement for airworthiness verification and an important means of fatigue-life assessment and safe-service evaluation of wing–body connection joints.

In terms of the macroscopic laws and microscopic mechanisms of fatigue crack propagation in titanium alloys, Jiang Tao et al. [3], Li Bingqiang et al. [4], Wang Bohan et al. [5], Gao Ning et al. [6], and Xu Xiangsheng et al. [7] studied the (ultra) high-cycle fatigue failure mechanism of TC4 titanium alloy. The results showed that different fatigue load stress ratios, different forging temperatures, and external damage will affect the fatigue damage mechanism of TC4 titanium alloy. Zhao Rongguo et al. [8] analyzed the cumulative damage and fatigue life of TC25 titanium alloy at room temperature under high-cycle fatigue, and the results show that the fatigue life of TC25 titanium alloy increases with the increase of the stress ratio under constant maximum stress. Liu Yu et al. [9] investigated the fatigue crack growth and growth behavior of IMI834 titanium alloy under high-cycle and ultrahigh-cycle fatigue, and the study showed that the IMI834 titanium alloy has a high fatigue strength under high-cycle and ultrahigh-cycle fatigue loading, the IMI834 titanium alloy has high fatigue strength, and there are two main crack initiation modes: surface initiation in short-life condition and internal initiation in long-life condition. Shang Guoqiang et al. [10] investigated the effect of three microstructures, bistatic, basketweave, and lamellar, on the high-cycle fatigue properties of TB17 titanium alloy. Zheng [11] studied the fatigue crack growth behavior of Ti-6Al-4V-ELI titanium alloy under different stress ratios and environments (air and brine), and the results showed that corrosive environments and high-stress ratios increased the rate of fatigue crack growth, especially in the stable stage of crack growth. Lei [12] investigated the microscale damage development and crack growth characteristics of TA15 titanium alloy with the tristate organization of isometric α (αp), lamellar α (αl), and β-phase transition matrix α (βt), and discussed the relationship between the fracture toughness of the tri-state organization and the microstructural parameters based on this study. Li [13] used two-stage annealing to heat TA29 titanium alloy forgings to investigate the fatigue crack growth behavior of the alloy at room temperature, 400 °C, 500 °C, and 600 °C and analyzed the fatigue fracture morphology of the specimens. Benedetti [14] investigated the effect of microstructural gradient Ti-6Al-4V and Ti-6242 titanium alloys on the resistance to fatigue crack growth; Ueki [15] used miniature tight tensile specimens with a single-cluster structure at the crack tip to investigate the mechanism of fatigue crack growth in the laminar cluster of Ti-6Al-4V alloys.

Fatigue tests and fatigue strength assessment of typical joint details of aviation structures are also crucial to ensure the safety of aviation vehicles. Zhang Lingyun [16] and others conducted bending fatigue experiments on Ti-3Al-2.5V titanium alloy unflared conduit joints, which showed that the high-cycle fatigue damage of the conduit joints consists of the fatigue source region, growth region, and final fracture region, and the fracture mechanism is caused by the surface slippage mechanism. Fan Junling [2] and others analyzed the fatigue fracture failure mechanism of bolted joint details of aircraft metal structures and showed that the fatigue crack initiation of bolted joints occurs at the stress concentration on the lower surface of the bolt holes in a multi-source mode. Ziqian et al. [17] conducted an experimental study on tensile fatigue properties for composite metal joints, which showed that the fatigue damage mode of composite/metal mechanical joints is closely related to the magnitude of the load level, and the damage of the metal structure is more likely to occur below a certain load level. Qin Zhengqi [18] and others carried out a study on the fatigue damage characteristics of countersunk hole bevels and the fatigue abrasion behavior of hole perimeters at low temperatures for the three-point bending fatigue strength of countersunk bolt-jointed composite members. Narita [19] designed fatigue strength experiments of titanium alloys to investigate the effect of micro-nano-scale wave structure on the fatigue strength of β-type titanium alloys. Soyama [20] designed experiments for the fatigue strength of titanium alloy Ti-6Al-4V and investigated methods to improve the strength of titanium alloys.

The initiation life, propagation law, and simulation prediction of fatigue cracks in metal materials are also popular research directions. Research in this area mainly focuses on the verification and application of numerical simulation methods for crack initiation and the influence of different types of load spectra on crack propagation life. Liang Jiaming et al. [21] proposed an image-driven model based on a spatiotemporal neural network (STNN) and conducted a crack-growth-prediction study based on aluminum alloy material experiments. Liao Zhen et al. [22] investigated the evolution of fatigue short cracks using a genetic wavelet neural network. Pan Shaozhen et al. [23] proposed a new method to predict crack growth curves based on Bayesian theory under a random load spectrum using Walker’s formula. Chen Xin et al. [24] proposed a four-parameter stochastic fatigue limit model for reliability design, which can achieve ultra-high-cycle fatigue stress-life curve processing for small sample data. Li [25] conducted ultra-long-life fatigue experiments at 106–109 cycles under asymmetric loading, elucidated the sub-surface facet-induced crack nucleation behavior of two α-β titanium alloys, and proposed a theoretical method for describing the strength relationship based on the microstructure. Chi [26] investigated the fatigue behavior of TC17 alloys with surface imperfections and developed a model to correlate the effect of flaws on fatigue strength.

It can be seen that the fatigue strength assessment of titanium alloy materials and the fatigue strength design of typical aircraft joints have been the focus of scholars. The experimental objects in the above study are partly standard bar experimental parts, which can provide a certain reference for the fatigue fracture analysis of titanium alloy joints but cannot directly react to the fatigue failure mechanism of titanium alloy joints’ experimental parts; the other part is multi-nail or other connection structures, which have great design differences with wing–body connection joints. In real aircraft structure, the wing–body connection joint is an important main bearing structure, and relevant experiments must be carried out to verify its fatigue strength, but due to the great difficulty of the experiments, the long duration cycle, and the huge consumption of manpower and material resources, the relevant reports are seldom seen. Therefore, in this paper, for a certain type of aircraft titanium alloy wing–body connection joint, the relevant fatigue test was designed, and the repeatability and reliability of the test apparatus were verified. After the fatigue test, the fatigue fracture was observed by scanning electron microscope (SEM), and the characteristics of the crack initiation region were discussed. The fatigue fracture mechanism of the joint was investigated, and the influence of microstructure on crack growth was analyzed. An engineering method was also used to quantitatively analyze the fatigue crack growth behavior of the wing–body joint and to verify whether the design of the test joint meets the requirements of aircraft safety and reliability.

## 2. Experiments and Methodology

### 2.1. Experimental Subjects

**Figure 1:**
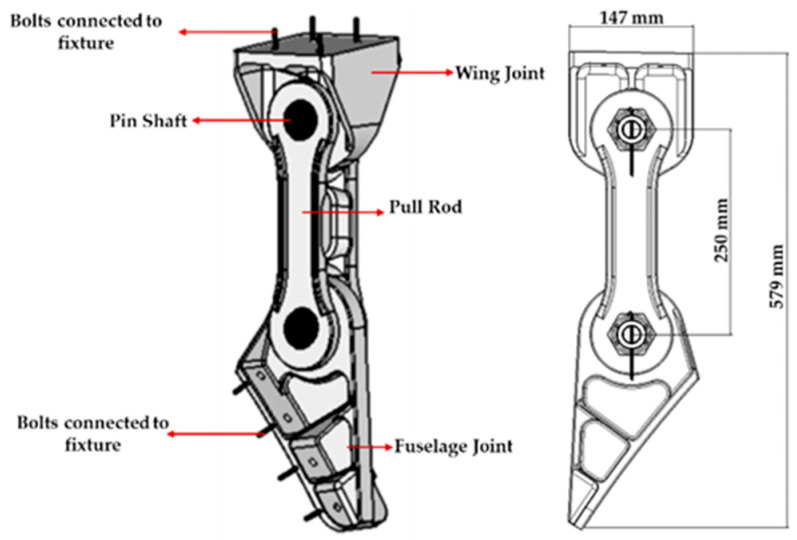
The diagram of the experimental piece configuration and the main dimensions.

The experimental object is the connection joint at the rear beam of the wing fuselage of the airplane. Each set of experimental parts includes the wing fuselage joint, the fuselage joint, the tie rods, the bolts connecting the joints and clamps, and the connecting pins between the joints and tie rods, and the materials of all the parts are Ti-6Al-4V. The structure and main dimensions of the experimental parts are shown in Figure 1.

### 2.2. Experimental Load Spectrum

**Figure 2:**
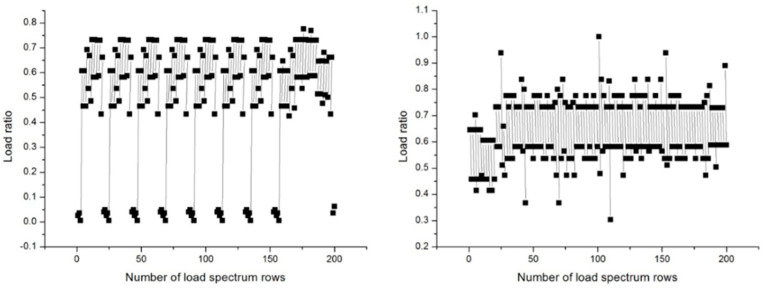
The diagram of the typical load spectrum.

To realistically simulate the flight conditions of the aircraft, this experiment adopts the random load spectrum for the titanium alloy wing–body connection joint. The load spectrum includes five types of flights, namely A, B, C, D, and E. The intensity of the five types of flights decreases step by step, with A being the most severe type of flight and E being the smoothest type of flight. Each flight randomly (pseudo-randomly) selects one of the five types of flights (A, B, C, D, E). Since the Class A flight spectrum is the most severe flight type, this paper conducts analysis on Class A flight loads and random load spectra. One block of the random load spectrum contains 6000 flight takeoffs and landings, totaling 195,794 load points, while the Class A load spectrum has a total of 544 load points. Both types of load spectra are tensile loads, and the maximum load is 159,531 N. Figure 2 gives the change rule of two typical blocks of 200 consecutive loads in the random load spectrum.

It is known from reference [27] that the fracture toughness K_IC_ of titanium alloy is 91.4 MPa.m^0.5^, the crack growth threshold Kth is 59.54 MPa.m^0.5^, and the ratio of the crack growth threshold to the fracture toughness is about 0.65. In the crack growth stage, the maximum load applied in this test is lower than the fracture toughness of the material itself. Therefore, it can be assumed that loads below the factor of 0.65 correspond to stress intensity factor values lower than the crack growth threshold. Based on this, this paper concludes that only loads greater than the 0.65 factor cause crack growth. The load distribution patterns of the two types of filtered load spectra with load ratios greater than 0.65 were statistically analyzed. The data with stress ratios greater than 0.65 were retained, while the data with stress ratios less than 0.65 were filtered out. The results are shown in Table 1.

### 2.3. Experimental Fixture Design

**Figure 3:**
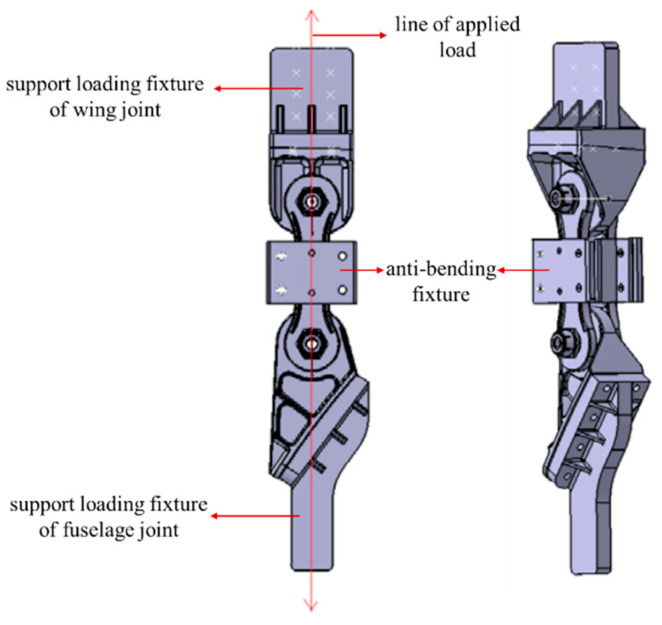
The diagram of the experimental support scheme.

To accurately simulate the actual loading of wing–body joints on the aircraft, the wing–body joints and fuselage joints were designed to support the loading fixture. One end of the support loading fixture was the mating–drilling connection with the wing–body joints and the fuselage joints, respectively, and the other end was the loading end, which was clamped in the experimental machine for the experiments. To avoid unintended bending of the pull rod during the experiment, an anti-bending fixture was designed (the anti-bending fixture was removed after it was found that no unintended bending was generated by analyzing the strain data in subsequent experiments). The line of applied load force was ensured to pass through the axis of the pull rod during the experiment. The experimental support scheme is shown in Figure 3. The experiments were carried out on a standard fatigue-testing machine (INSTRON/1000kN) (Instron Engineering Corporation in Boston, MA, USA). The fatigue-testing machine has a built-in displacement sensor, which can measure and output the displacement change of the test piece under the fatigue load spectrum in real time. The displacement sensor built into the standard fatigue testing machine monitors the displacement value and determines whether the test piece has a fatigue fracture. The displacement data generated in the experiment are collected and visualized by the host computer to achieve the effect of monitoring the fracture of the wing–body connector. The support loading fixture material is 30CrMnSi, and the anti-bending fixture material is 45# steel.

### 2.4. Experimental Procedures

To ensure the accuracy of the experiment and to achieve the desired experimental purpose, the pre-test experiment, the strain measurement experiment, and the fatigue experiment (including the crack growth experiment) were planned sequentially. The purpose of the pre-test experiment was to check whether the experimental loading system and the measurement system were normal before the start of the formal fatigue experiment, and at the same time, to adjust the loading frequency to ensure that the experimental equipment operated smoothly and, after adjusting, to determine the experimental loading frequency of 10 Hz.

**Figure 4:**
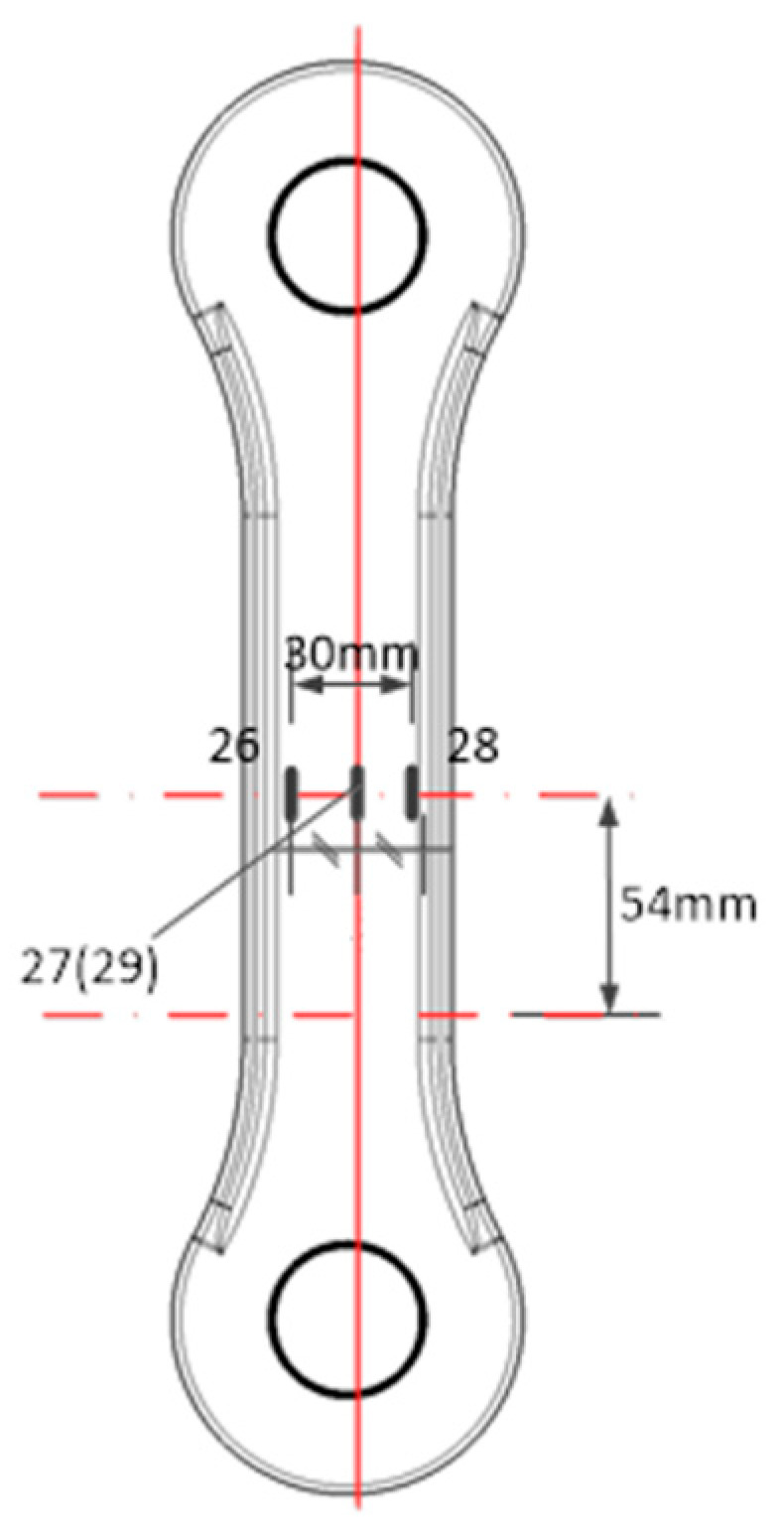
Schematic diagram of attaching strain gauges on pull rod.

The strain measurement experiment was a static tensile test, and a strain gauge sensor was used to characterize the load-bearing condition of the specified part of the specimen under the action of the load. The main purpose of the strain measurement experiment was to analyze the accuracy/symmetry and repeatability of the experimental loading, so as to ensure the accuracy of the experiment. The location of the strain gauges pasted in the experiment is shown in Figure 4. It was pasted on the front and back sides of the tie rod with a clear force transmission path to obtain the load condition of the joint component. After that, the strain gauge was connected to the DH3820 multi-channel strain acquisition system to collect the strain data generated in the experiment. In the experiment, the maximum load was taken as the maximum value of the Class B load spectrum, 133,655 N, and the load was loaded step by step, with a step difference of 10%. The load was measured step by step until it reached 100% of the maximum load and then unloaded step by step. The experiment was repeated 3 times. The experimental accuracy was evaluated by analyzing the repeatability, linearity, and symmetry of the strain data in the three experiments.

The purpose of the fatigue experiments was to obtain the fatigue life of the wing–body joint and to reveal the crack extension parameters. First, a random load spectrum was used to complete 400,000 flight take-offs and landings to verify whether the test piece met the design target. During the test, when cracks were found in parts other than the joint, new parts were replaced, and fatigue tests were continued to assess the joint. If cracks were found at the joint lug before 400,000 take-offs and landings, the crack extension test was immediately carried out. If no cracks were found at the joint lug after 400,000 take-offs and landings, it meant that the wing–body connection joint met the design life requirements. If no cracks were found at the joint after 400,000 take-offs and landings, a larger load (Class A flight load spectrum) was used to continue the test. If no cracks are found at the joint lug after 100,000 Class A flight load spectra, the ground–air–ground load was used to continue the test until the joint lug breaks, and then the test piece was disassembled to observe the specific location of the crack and analyze the fracture.

**Figure 5:**
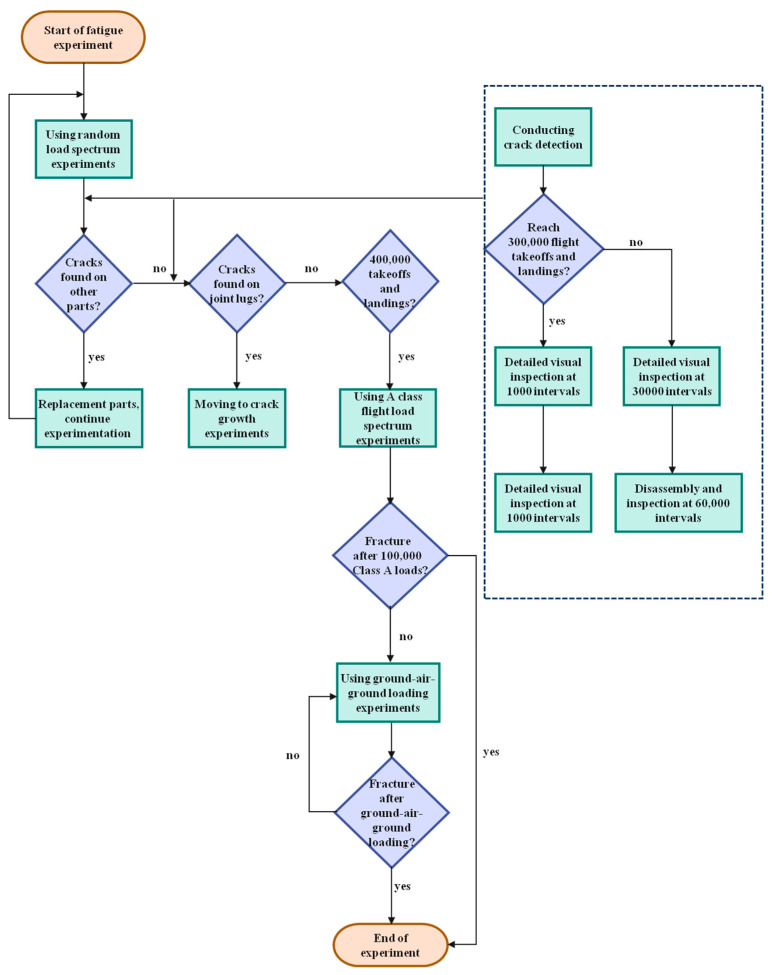
Fatigue experiment scheme and inspection interval flow chart.

Crack detection was carried out on the examination part during the fatigue experiment. The method was to disassemble the joints and pull rod connecting pins and then carry out nondestructive inspection of the examination parts by eddy current. The inspection intervals were as follows: before 300,000 take-offs and landings, a detailed visual inspection was carried out every 30,000 times, and the experimental piece was disassembled and inspected every 60,000 times; after 300,000 flight take-offs and landings, detailed visual inspection was carried out every 1000 times, and the experimental piece was disassembled and inspected every 10,000 times. Crack inspection was no longer performed after the design target was reached. The fatigue experiment scheme and the flow of inspection intervals are shown in Figure 5.

After the experiment, a scanning electron microscope was used for the broken wing–body connection joint to perform macroscopic and microscopic analysis on the specimen after fatigue damage. The fractures of the specimens at different crack extension stages were placed under the electron microscope lens, and the surface of the failed specimens was photographed by a high-energy electron beam to obtain the morphological data at different crack extension stages, to understand the specimen failure process and analyze the specimen failure mechanism.

To further observe the overall picture of the broken wing–body connection joint, a stereo microscope was used to further observe the worn parts of the ear hole. The size, shape, and surface morphology of the specimen were obtained by photographing the side morphology of the fracture to analyze the cause of the specimen cracking. In addition, the results of the stereo microscope supplemented the failure process and failure mechanism of the specimen.

### 2.5. Experimental Accuracy

**Figure 6:**
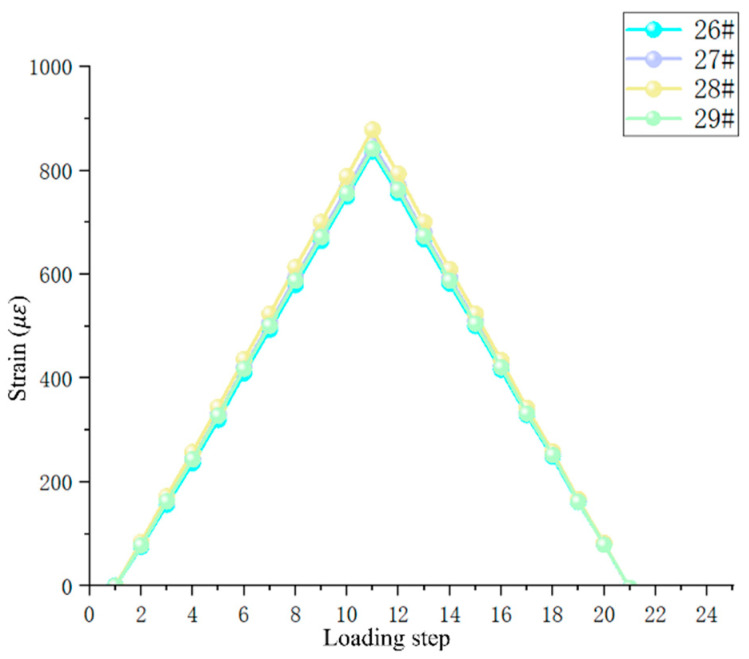
Symmetry analysis of strain measurement data.

The accuracy of experimental instruments such as sensors was the key to obtaining correct experimental results. In order to ensure the correctness of the experimental data, the data of the symmetrical strain test experiment were used to verify the accuracy of this experiment. The strain data collected in the strain measurement experiments were analyzed for errors. The data of the first strain measurement experiment are shown in Figure 6, which shows that the linearity of the experimental loading process and unloading process was fine, and the strain values corresponding to the same load had good symmetry. The data of axisymmetric strain (26#, 28#) in the surface of the pull rod and the data of symmetric strain (27#, 29#) in the front and back surfaces were analyzed, and the maximum error was 4.94% and 1.19%, respectively.

**Figure 7:**
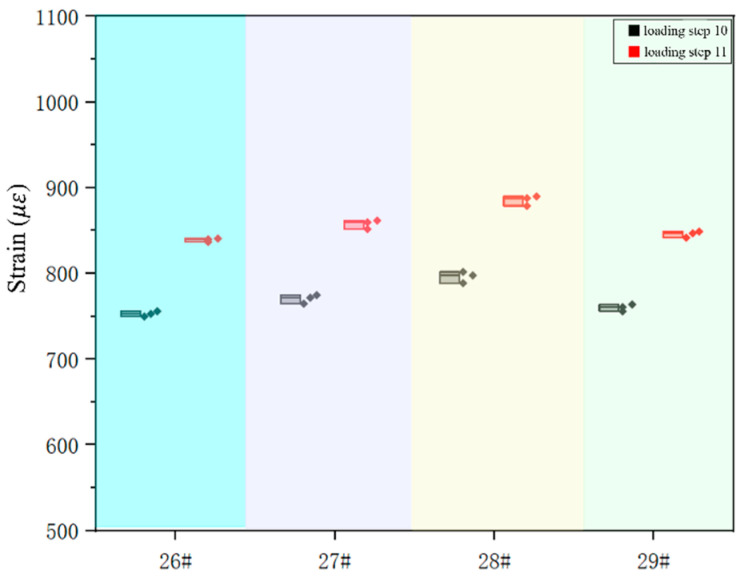
Repeatability analysis of strain measurement data.

This experiment was a fatigue experiment, and the repeatability of the experiment is also a key indicator for obtaining correct data. To verify the reproducibility of the experiments, independent repeat experiments were conducted in this study. The focus was on the strain response at peak stress, since strain at peak stress was the key variable affecting the experimental results. Figure 7 shows the results of the visualization of the strain data under peak stress, revealing a high degree of consistency in the strain response of the data from the three experiments, and no outliers were observed throughout the testing process, thus confirming the excellent repeatability of the 26#, 27#, 28#, and 29# strain gauge tests. By accurately calculating the error for a specific loading step, it was found that the 28# strain gauge had the largest error of 0.5% in loading step 10, an error value that was within an acceptable range, further solidifying the reliability of the experimental results.

### 2.6. Crack Growth Methodology

In the aerospace field, the fatigue crack growth life of wing–body connection joints as a proportion of the full life cycle is an important criterion for assessing the structural characteristics of wing–body connection joints. Therefore, in this paper, the fatigue crack growth life of the joint was calculated to assess the damage tolerance characteristics of the component, so as to judge whether the joint structure met the design requirements.

Firstly, in the ideal case, for every cycle of the load, the crack growth takes one step forward and forms a fatigue strip on the fracture [1,28,29], since the spacing of the fatigue strips can reflect the growth rate of the crack at the microscopic scale level [29]. Therefore, in this paper, the fatigue strips appearing in the fracture of an aircraft titanium wing–body connection joint are used to make an inverse deduction, so as to predict the fatigue life of the wing–body connection joint under the real operating conditions of the aircraft.

There exist many models to describe fatigue cracks based on fracture mechanics [30]. In this paper, the Paris formula is used to describe the crack growth rate. The following relationship between fatigue crack growth rate and stress intensity factor can be established according to the Paris formula [31]:

(1) $$\frac{da}{dN}=c{\left(\Delta K\right)}^{m},$$

where *c* and *m* are material constants, a is the crack length, and Δ*K* is the stress intensity factor magnitude.

According to the literature [32], it is known that the stress intensity factor is a function of the crack, and therefore, the Paris formula can be expressed by the following equation:

(2) $$\frac{da}{dN}=\alpha a^{\beta },$$

where α and β are constants.

Integrating Equation (2), the fatigue crack growth life model can be constructed as shown in Equation (3).

(3) $$N=\int_{a_{\min}}^{a_{\max}}\frac{1}{\alpha a^{\beta }}da=\frac{1}{\left(1-\beta \right)\alpha }a^{1-\beta },$$

where *a*_max_ and *a*_min_ are the maximum and minimum values of the crack length, respectively.

## 3. Results and Analysis

### 3.1. Analysis of Fracture Macro-Mechanisms

In this study, fatigue experiments were conducted on a titanium wing–body connection joint at the rear beam of an aircraft, which lasted until the joint fractured. During this process, the wing–body joint experienced 400,000 takeoffs and landings with random spectra, 100,000 takeoffs and landings with Class A loading spectra, and 339,043 cycles with ground–air–ground loading spectra.

**Figure 8:**
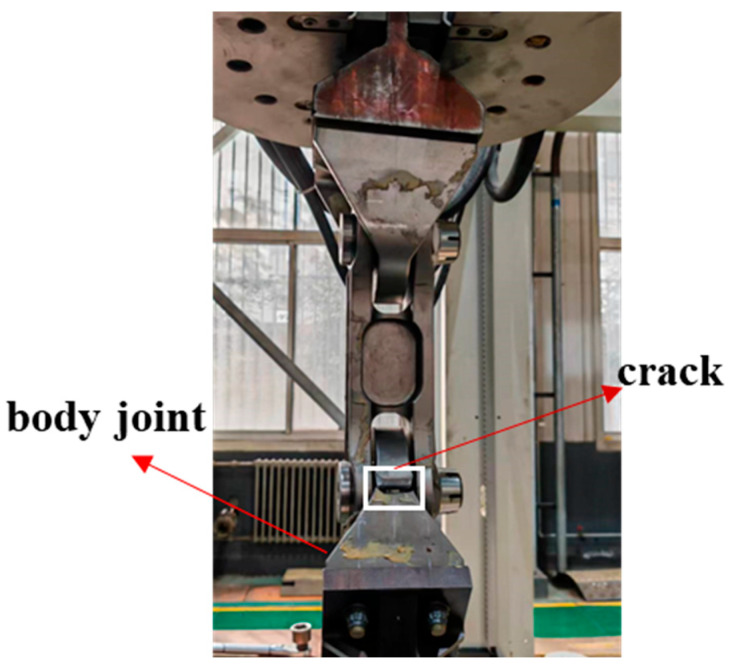
Diagram of the joint fatigue fracture.

Figure 8 illustrates the fatigue fracture results of an aircraft wing–body joint, clearly revealing that the fracture site is located at the fuselage joint lugs. To gain a deeper understanding of the fracture mechanism, a systematic failure analysis of the fractured specimen was subsequently carried out to reveal the root cause of the fracture and its impact on the structural integrity.

**Figure 9:**
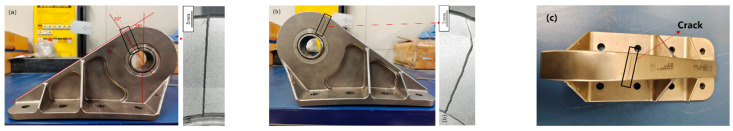
Diagram of crack location and macroscopic shape of the experimental piece. (**a**) a side view of the wing-fuselage connector, with a stereoscopic microscope diagram showing the fracture location on the right; (**b**) another side view of the wing-fuselage connector (the back of (**a**)), with a stereoscopic microscope diagram showing the fracture location on the right; (**c**) a top view of the wing-fuselage connector.

Figure 9 is a schematic diagram of the crack in the wing–body connection joint after fracture, showing the shape and location of the titanium wing–body connection joint experimental piece throughout the failure process. From Figure 9, it can be found that the fracture occurred near the transition between the circular segment and the straight line of the experimental piece. The maximum principal stress around the crack was calculated to be 450 MPa by the finite element method. Meanwhile, it can be found that the crack direction is close to the radial direction of the lugs on side 1, shown in Figure 9a. By observing and measuring the crack direction, it can be found that the shape of the crack is a straight line, the angle between the crack direction and the radial direction at the arc–straight line transition is about 10°, and the angle between the crack direction and the vertical direction of the experimental piece when it is laid flat is about 25°. On side 2 (back of side 1), shown in Figure 9b, the crack growth along the straight line was deflected after a certain distance. On the surface of the top view shown in Figure 9c, the crack takes on an arc shape.

**Figure 10:**
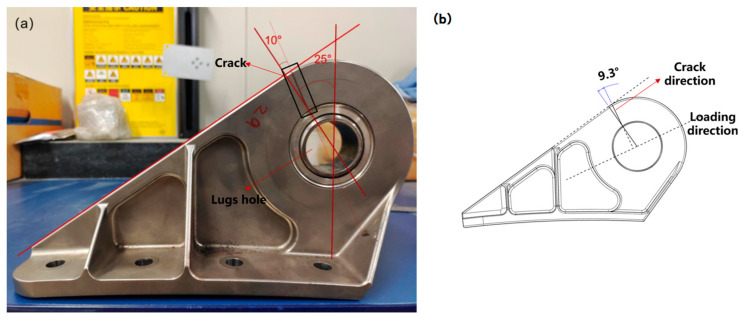
Diagram of the direction of loading force and crack direction. (**a**) physical picture of the experimental piece; (**b**) plan view of the experimental piece.

Firstly, the direction of crack growth shown in Figure 9a was carefully analyzed, and it was found that there exists an angle of about 9.3° between the direction of the loading force and the radial direction at the circular–straight line transition, as shown in Figure 10. It can be known from the relationship shown in Figure 10 that there is a perpendicularity between the path of crack growth and the experimental loading force line, which provides a key perspective for understanding crack behavior. Meanwhile, the perpendicularity points out the direct correlation between the direction of crack growth and the direction of the applied loading force, which is consistent with the pattern of fatigue crack growth.

**Figure 11:**
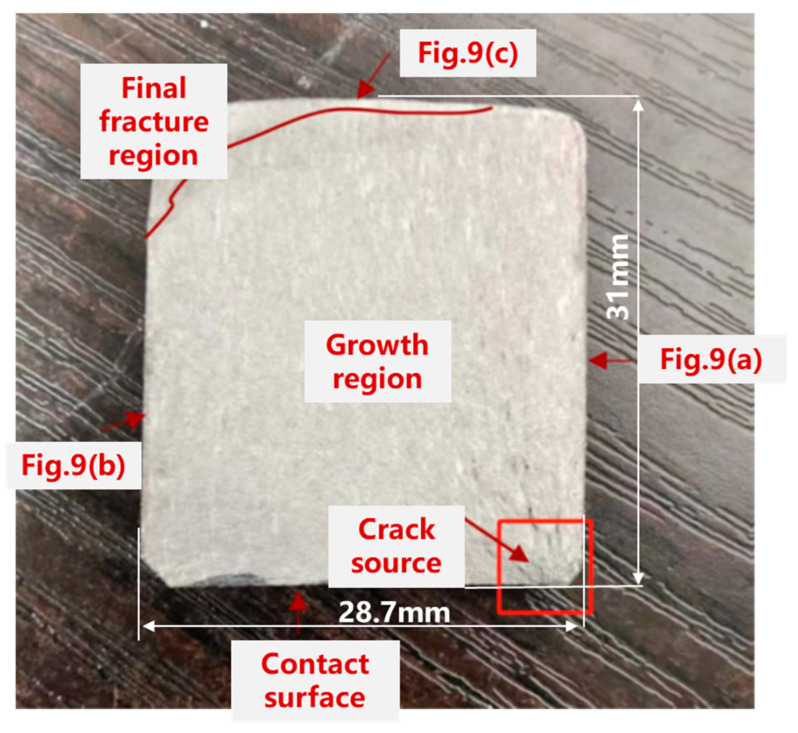
Macroscopic morphology of the fracture of the experimental piece.

After that, the fracture was removed for electron microscopy analysis by the wire cutting method, and the macroscopic morphology of the fracture is shown in Figure 11. The correspondence between the four edges of the fracture in Figure 11 and each side of the experimental piece in Figure 9 is as follows: the right side of Figure 11 is Side 1 shown in Figure 9a; the left side of Figure 11 is Side 2 shown in Figure 9b; the upper side of Figure 11 is the surface of the top view shown in Figure 9c; and the lower side of Figure 11 is the contact surface of the hole of the lugs and the pin. Based on the fracture morphology in Figure 11, the fracture surface of the wing–body connection joint can be roughly divided into the following three characteristic regions: fatigue source region, growth region, and final fracture region. The growth region occupies most of the area, and the growth region is relatively flat and smooth, while the source region and the final fracture region are relatively rough.

The fatigue source area is the crack initiation area. It can be observed from Figure 11 that fatigue crack initiation occurs on the contact surface between the lug hole and the pin, which is less than 1 mm away from the side shown in Figure 9a. At the same time, the main direction of crack propagation is close to the radial direction of the lug hole; that is, it extends from the contact point between the lug hole and the pin to the top view surface, shown in Figure 9c. The main reason for the initiation of cracks in this area is that this is a geometric discontinuity area, and this geometric discontinuity will cause stress concentration in this area [33,34]. When the stress exceeds the fatigue strength of the wing–body connection, it will cause crack initiation. Secondly, this geometric discontinuity will cause or aggravate small vibrations in this area [35,36]. In this case, the lug hole and the pin will experience micro-motion wear. Finally, the wing–body connection initiates cracks under the combined action of micro-motion wear and stress concentration. In addition, many studies have shown that the failure of the connection structure often occurs in these geometric discontinuity areas [37,38]. On the other hand, Figure 9a also shows the direction of crack propagation. This is mainly because the maximum principal stress direction in this test is perpendicular to the loading force line, and the direction of crack propagation in the wing–fuselage connection in Figure 9a is the direction of the maximum principal stress direction. This result also proves the correctness of the test and analysis methods again.

As the crack grows, the fatigue crack enters the crack growth period. In the early stage of crack growth, the crack will grow in a straight line from the crack source along the principal stress direction. As the crack grows, due to limitations such as component size, the cracks near the side of the source region will first grow to the end of the joint. In the case of cyclic loading, the crack growth at the side away from the source region will appear after a while along the straight line growth due to the limitation of the dimensions, as shown in Figure 9b. At the same time, it can be seen from the crack detail diagram shown in Figure 9a that the crack morphology in the linear expansion stage of this side is very regular, while the linear expansion stage of the side shown in Figure 9b shows a step-like dislocation, which directly indicates that there are defects in the side material shown in Figure 9b, causing the crack to encounter microscopic obstacles inside the material, such as grain boundaries, inclusions, or phase boundaries, thereby changing the crack expansion path [39,40]. At the same time, these defects further cause the crack on this side to deflect during expansion.

In the final fracture region, the crack growth is very fast because it is controlled by the fracture toughness and local stress state of the material. Since the growth in the final fracture region is rapid and unstable, the crack morphology is no longer the straight-line morphology of the growth period but an irregular shape, as shown in Figure 11. Since most of the surface of the top view shown in Figure 9c is the final fracture region, the crack thus exhibits an irregular shape, i.e., arc-like.

**Figure 12:**
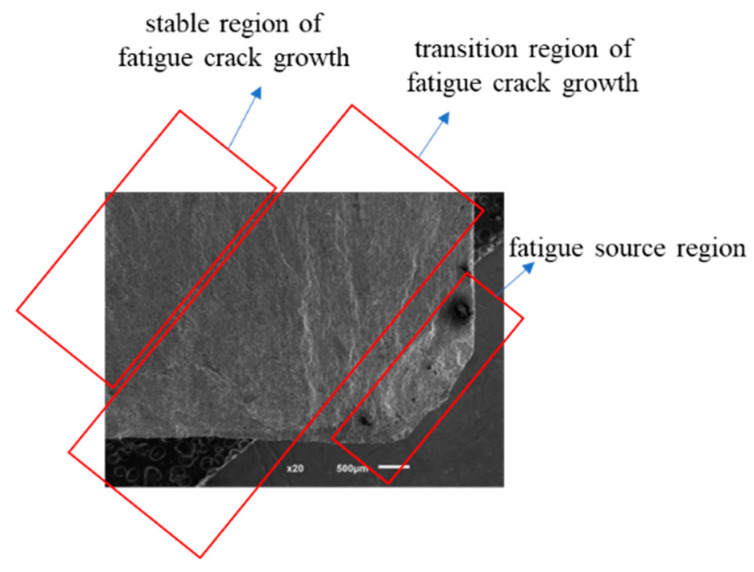
SEM electron micrograph of the fracture.

To deeply investigate the growth mechanism of fatigue cracks, this study used an electron microscope to scan and observe the fracture surface, as shown in Figure 12. The observation results show that the crack growth region in the fatigue specimen is relatively smooth and exhibits obvious polishing features, which is the result of mutual contact and friction of the material surfaces under cyclic loading. The adjacent crack initiation region is relatively rough, which is mainly due to the repeated tensile motion of the upper and lower sections of the specimen during the fatigue crack growth process. At the junction of the crack initiation and growth regions, several fine radial lines can be observed, which have neither the roughness of the crack initiation region nor the smoothness of the growth region, indicating the early stage of crack growth. This finding reveals that the transition of cracks from the initiation region to the growth region is not a mutation process, but a continuous process of gradual evolution, in which both the morphology and the growth mechanism of cracks are constantly changing. Therefore, this particular region is named the transition region of fatigue crack growth. Through this region, the crack growth behavior will gradually converge to a stable state, which provides an important perspective for understanding the fatigue crack expansion mechanism and helps to predict the crack behavior under actual working conditions, which is of great significance for improving the fatigue life prediction accuracy of structures.

### 3.2. Analysis of Fracture Micro-Mechanisms

**Figure 13:**
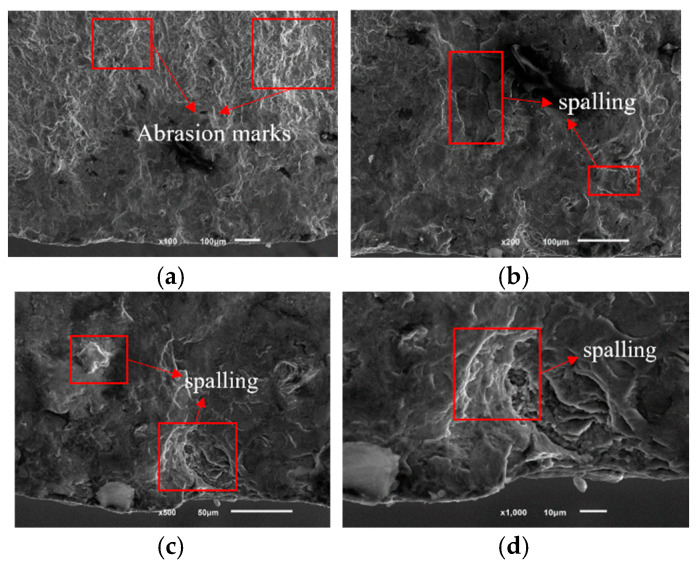
SEM electron micrograph of the fatigue source region. (**a**) SEM electron microscope photo at 100 times; (**b**) SEM electron microscope photo at 200 times; (**c**) SEM electron microscope photo at 500 times; (**d**) SEM electron microscope photo at 1000 times.

Figure 13a–d show SEM electron micrographs of the fracture source region of the wing–body joint at different magnifications. From the comparison of Figure 13a,b, the abrasion marks in the contact area and the spalling pits formed during the abrasion process can be observed. Figure 13c,d further demonstrate the morphology of the spalling pits, through which it can be found that the formation of the spalling pits was due to the phenomenon of micromotor fatigue caused by small-amplitude relative sliding between the contact surfaces, which led to a gradual loss of material to form the spalling [3]. In the initial stage of micromotion fatigue, cyclic contact stresses were generated on the contact surfaces due to small-amplitude relative sliding, and especially in the localized stress concentration areas, the titanium alloy material of the wing–body joints started to fracture and form initial cracks [33,41]. Griffith’s theory of fracture states that crack initiation occurs when these stress concentrations exceed the fracture toughness of the material, as shown in Figure 11. In addition, the frictional heat and oxidation generated during the micromotion process further accelerated the crack nucleation, and these factors together led to the initiation and growth of cracks in the source region.

**Figure 14:**
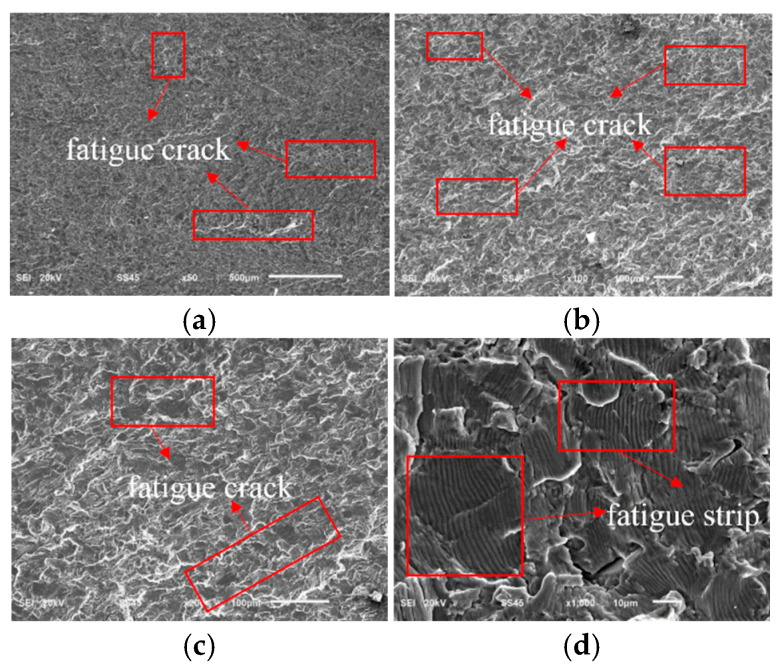
SEM electron micrograph of fatigue growth region. (**a**) SEM electron microscope photo at 50 times; (**b**) SEM electron microscope photo at 100 times; (**c**) SEM electron microscope photo at 200 times; (**d**) SEM electron microscope photo at 1000 times.

With the crack growth, the wing–body joint entered the stable growth stage of the crack. The stable crack growth stage was observed by electron microscope images at different magnifications. Figure 14a,b reveal the fatigue cracks at the crack fronts, which are the main features of crack growth under cyclic loading. With the growth of magnification, Figure 14c,d further demonstrate the details of the fatigue fracture traces, including fatigue steps and crack branches, and these microscopic features confirm that fatigue fracture is the main failure mechanism in the crack growth region [42]. At this stage, the crack growth behavior follows Paris’ law; i.e., the rate of crack growth is proportional to the square root of the stress intensity factor. Under external cyclic stress, the crack growth continues based on the existing crack source, forming the morphological features observed in Figure 14. Also, in this region, since the crack growth is due to cyclic loading, each crack growth is in the direction of the maximum principal stress of the cyclic stress. Each cycle may cause a small growth of the crack front, which ultimately leads to the straight-line pattern of the macroscopic morphology during the stable crack growth period [28,29], as shown in Figure 11. However, the crack growth path and rate are not only affected by the cyclic loading but also by the material microstructure, grain size, and inclusions. As a result, the step-like crack shape in Figure 9b also occurs during the crack growth period [39,40]. In addition, the fatigue resistance of the material and loading conditions also have a significant effect on the crack growth rate. When the crack growth reaches a later stage, the stress concentration will lead to a significant increase in the crack growth rate until the crack reaches a critical size, leading to the final fracture of the structure. These observations provide important microscopic evidence for understanding the failure mechanism of wing–body joint components under fatigue loading.

**Figure 15:**
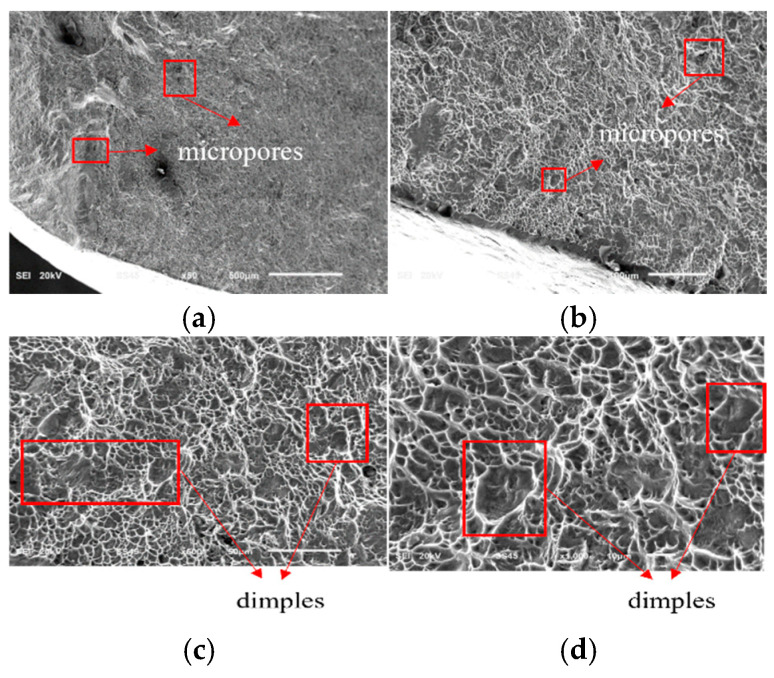
SEM electron micrograph of fatigue final fracture region. (**a**) SEM electron microscope photo at 50 times; (**b**) SEM electron microscope photo at 100 times; (**c**) SEM electron microscope photo at 500 times; (**d**) SEM electron microscope photo at 1000 times.

Figure 15 shows SEM electron micrographs of the final fracture region of the wing–body joint. These images record the final stage before fracture. In the final fracture region, the crack growth occurs rapidly due to reaching the critical size, leading to the instantaneous fracture of the material. Figure 15a,b show the microscopic features of the fracture surface, from which the micropores on the surface of the material can be detected, whereas the aggregation of micropores and cavitation before fracture are typical features of ductile fracture, and these phenomena indicate that the material undergoes significant plastic deformation before fracture [43,44]. In addition, the fracture process is accompanied by the formation of tear ridges, which are microstructures created by the plastic deformation of the material during the fracture process. It is this extensive plastic deformation and damage that leads to the rapid growth of fatigue cracks and the production of irregular shapes, as shown in Figure 11 [28]. With increasing magnification, Figure 15c,d show more clearly the morphology of the dimples which are the result of localized plastic deformation of the material in regions of high-stress concentration and subsequent fracture at grain boundaries or inclusions [1,29,43]. Thus, in the final fracture region, the fracture mechanism changes from fatigue crack growth to instantaneous ductile fracture. Fracture at this stage occurs as a result of rapid fracture due to the inability of the material to withstand the applied load and is usually accompanied by a rapid release of energy.

**Figure 16:**
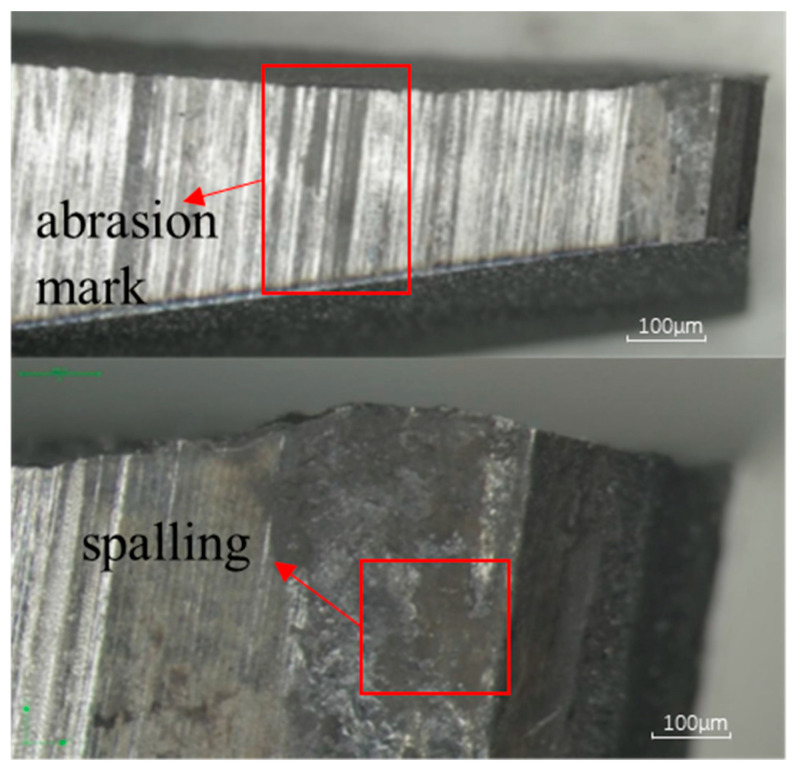
Stereo microscopic results of the fracture.

To further verify the crack growth mechanism, in this study, a stereo microscope was used to observe the fracture side, i.e., the morphology of the contact surface between the lug hole and the bearing. Figure 16 illustrates the observation results, in which it is can be seen that the contact surface, especially near the crack source region, retains traces caused by friction as well as surface metal peeling. These features indicate that small vibrations between the lug hole and the bearing due to micro-motion fatigue effects are the dominant factor in crack formation. Therefore, the observation results of the stereo microscope further support the analysis of the cause of crack initiation in the wing–body joint.

### 3.3. Result of Fatigue Crack Growth Life of Wing–Body Connection Joints

Estimating the fatigue crack growth life of wing–body connection joints as a proportion of the full life cycle is important for assessing the damage tolerance characteristics of wing–body connection joints.

**Figure 17:**
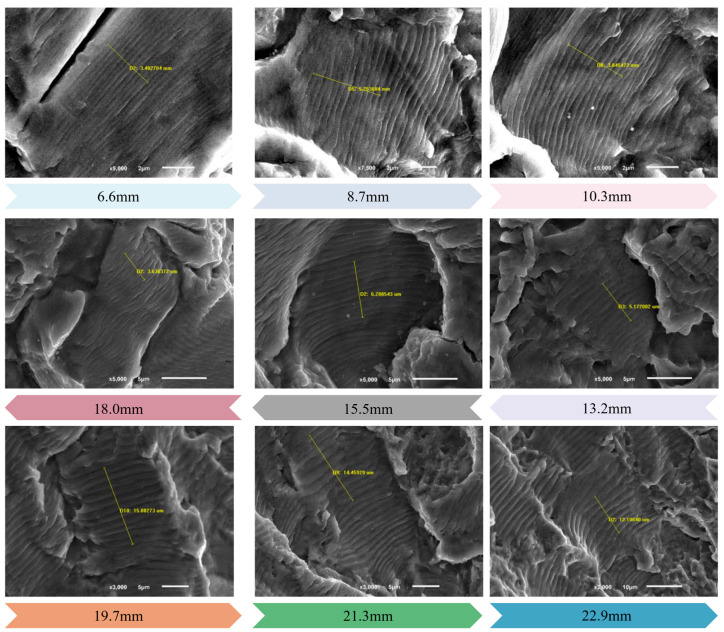
Fatigue stripes at different crack lengths in the crack growth region.

The fatigue crack growth rate in the primary source region was quantitatively measured and characterized using scanning electron microscopy. Since the crack growth rate da/dN is the distance the crack grows forward every cycle, its value is equal to the width of a fatigue strip. However, in the actual measurement process, to reduce the error, the average value of the width of multiple fatigue strips is usually taken as the crack growth rate. The crack diagram in Figure 17 shows the quantitative measurement of the number and spacing of fatigue strips during the stable crack growth process. It can be found that with the increase of the fatigue crack length, the fatigue stripe spacing gradually increases under the same number of load cycles, and the crack growth rate also increases, which also reflects the increasing stress intensity factor.

The crack growth rates at different crack lengths were calculated by measuring the fatigue strip widths, and the results are shown in Table 2.

**Figure 18:**
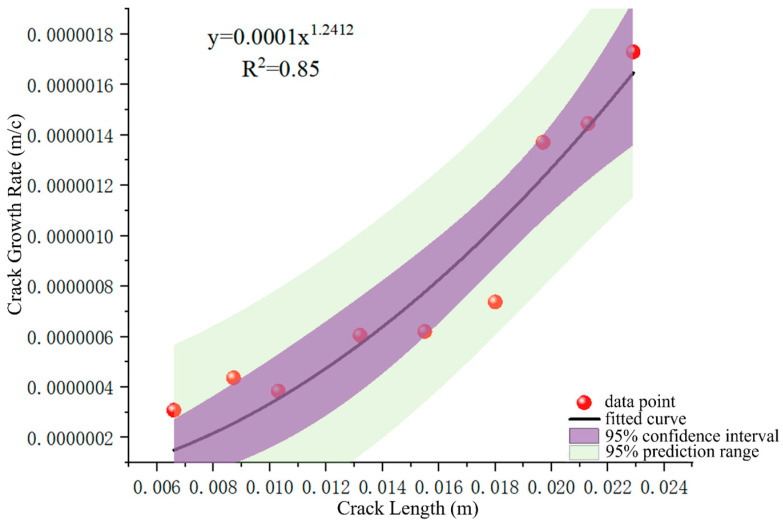
Relationship between crack growth rate da/dN and crack length *a*.

According to reference [32], the crack growth rates obtained from the electron microscopy quantitative measurements in Table 2 are in the stable crack growth period. Therefore, the crack growth analysis using Paris’ law has good applicability. Based on the measured and calculated results in Table 2, the functional relationship between the fatigue crack length a and the crack growth rate da/dN was established using the least-squares fitting method, as shown in Figure 18. With this fitting result, the crack growth behavior in practical applications can be effectively predicted and evaluated, thus providing scientific guidance for the maintenance and safe operation of aircraft structures.

According to the fitting results in Figure 18, we can get α = 0.0001 and β = 1.2412. Using Equation (2) to invert the crack growth life, assume that the initial crack length of the wing–body joint is 1.25 mm [27], and invert the critical crack length of 21.9 mm according to the residual strength theory. In the range of crack growth length of the segment, the fatigue can be obtained by substituting the calculation results into Equation (3) for the calculation of crack growth life:

(4) $$N=\int_{a_{min}}^{a_{max}}\frac{1}{1\times {\left(10\right)}^{-4}a^{1.2412}}da=103,687,$$

Since two loadings constitute a cycle in the experiment, the number of load spectrum loadings is

(5) $$N_{e}=2N=207,374,$$

where N_e_ is the number of load spectrum loading.

The number of payload rows N_s_ = 4,313,276 experienced by the wing–body joint in the full life cycle can be calculated from the experimental data. The fatigue crack growth life as a percentage of the full life cycle is

(6) $$\delta =\frac{N_{e}}{N_{s}}=\frac{207,374}{4,313,276}=4.81\%,$$

where δ is the fatigue crack growth life as a proportion of the full life cycle, and N_s_ is the test result.

It can be found through calculation that for this titanium alloy wing–body joint, the crack initiation life accounts for 95.19% of the full life cycle and is much larger than the design requirement of 400,000 takeoffs and landings, which indicates that the structural design of this test is a conservative design, which is in line with the requirements of the operational safety of the aircraft.

## 4. Conclusions

This paper took the titanium wing–body connection joint at the rear beam of a certain aircraft as the research object, simulated the real flight conditions of the wing–body connection joint, designed the full-scale fatigue test of the wing–body connection joint, analyzed the failure mechanism using the test results, and obtained the following conclusions:

1. The full-scale fatigue test of the wing–body connection joint designed in this paper has proved to be feasible, and capable of accurately simulating the actual flight conditions and effectively obtaining key test data. The implementation of the pre-test further verified the reliability of the loading system and the measurement system, providing a guarantee for the accuracy of the experiment.
2. This study identifies that the weak link of the wing–body connection joint is located at the lug hole and finds that the fracture originates in the transition region between the circular arc segment and the straight line. The crack growth path is nearly perpendicular to the experimental loading force line, indicating that the direction of the maximum principal stress is the main driving force for crack growth. During crack growth, side cracks away from the source region fluctuate due to size constraints, leading to path deviation. At the same time, the crack encounters internal microscopic barriers in the material during growth, such as grain boundaries, inclusions, or phase boundaries, all of which alter the crack growth path. The transition period of cracks from the region of initiation to growth is a continuous process of gradual evolution, in which the morphology of the fatigue specimens gradually changes from rough to smooth, revealing the microscopic mechanism of fatigue crack growth.
3. The geometric discontinuity between the lug hole and the pin leads to local micromotion fatigue, causing small amplitude relative sliding between the contact surfaces, which is the dominant factor in crack formation. In the growth stage, the crack is affected by external cyclic stress and continues to grow based on the existing crack source, forming fatigue steps and crack branches, which are the main features of fatigue damage. In the final fracture region, the wing–body joint mainly exhibits ductile fracture, with significant plastic deformation before fracture and the formation of dimples in the local plastic deformation region. The plastic deformation process is accompanied by the formation of tear ridges, and these phenomena indicate that the material undergoes complex microscopic changes before fracture.
4. According to the definition of the crack growth period of titanium alloy, the life of the crack growth period of the wing–body connection joint of the aircraft is calculated to be 207,374 loadings, accounting for 4.81% of the full life cycle of 43,132,776. The crack initiation life accounts for 95.19% of the full life cycle, far exceeding the design requirement of 400,000 landings and takeoffs, confirming that the structural design is on the conservative side and meets the requirements of aircraft operational safety.

## Figures and Tables

**Table 1:** Statistic analysis of distribution law of random load spectrum.

Type of Load Spectrum	Load Ratio	Number of Rows	Proportion %
random load spectrum	>0.65	59,570	30.4
class A load spectrum	>0.65	174	31.9

**Table 2:** Quantitative crack growth rate measurement results.

Number	Crack Length (mm)	Fatigue Strip Width (μm)	Number of Fatigue Bands	Crack Growth Rate (MPa.mm^0.5^)
1	21.9	12.11	7	1.73^−6^
2	21.3	14.46	10	1.45^−6^
3	19.7	15.08	11	1.37^−6^
4	18	5.17	7	7.39^−7^
5	15.5	6.21	10	6.21^−7^
6	13.2	3.64	6	6.07^−7^
7	10.3	3.85	10	3.85^−7^
8	8.71	5.25	12	4.38^−7^
9	6.59	3.4	11	3.01^−7^

## Data Availability

Data are contained within the article.
